# A profile of urban agricultural growers, organizations, their needs, and challenges in the Northeastern United States

**DOI:** 10.1371/journal.pone.0298831

**Published:** 2024-04-10

**Authors:** Matthew L. Richardson, John R. Taylor, Megan J. Thompson, Anusuya Rangarajan, Mamatha Hanumappa, Neith G. Little

**Affiliations:** 1 Center for Urban Research, Engagement and Scholarship, University of the District of Columbia, Washington, District of Columbia, United States of America; 2 Department of Plant Sciences and Entomology, University of Rhode Island, Kingston, Rhode Island, United States of America; 3 School of Integrative Plant Sciences-Horticulture Section, Cornell University, Ithaca, New York, United States of America; 4 Agriculture and Food Systems Program, University of Maryland Extension, Baltimore City Office, Baltimore, Maryland, United States of America; University of California Davis, UNITED STATES

## Abstract

Urban agriculture is increasingly valued as a strategy for improving quality of life in cities, but urban growers face challenges and often lack coordinated support from governments and the agricultural industry. We surveyed urban growers through an online survey, primarily in the Northeastern United States, to develop a profile of growers and associated organizations, assess the current state of urban agriculture, and determine how universities could help meet their needs. A total of 394 respondents completed the survey and most urban growers were white (non-Hispanic) and younger than 45 years old. Women and men were in almost equal proportion. Urban growers were well-educated, but most did not receive a degree in agriculture. Urban agriculture in our study area was dominated by relatively small non-profit organizations and home and community gardens were the most common types of organizations. Urban agricultural organizations want to improve environmental sustainability and socio-cultural conditions through food access and security, regardless of their tax status. Urban growers face diverse barriers and challenges and the most ubiquitous barriers and challenges reported by respondents were related to availability of land and long-term access in urban areas. Many respondents received low revenue or were operating at a net loss even though they reported diverse income streams. Respondents need a wide range of training, including in traditional agricultural topics as well as financial management and business trainings. Universities can play a key role in promoting urban agriculture by offering training and research. Workforce development is a large priority among universities, so urban growers should regularly be consulted, and the results shared with career and workforce development professionals and researchers in urban areas to identify training and research that meets the needs of stakeholders.

## 1. Introduction

Urban agriculture, farming within urban and peri-urban spaces [1], is increasingly valued as a strategy for improving quality of life in cities, with potential benefits for food security [2, 3], socio-economic resilience [4, 5], and ecosystem services [6–8]. Cities may have ample, suitable space to dramatically expand food production in backyards, in vacant lots, and on public land and rooftops. However, expansion faces several social, economic, and environmental challenges [9]. Urban agriculture initiatives may suffer from a lack of community, academic, and government support due, in part, to the perception that agricultural production is incommensurate with city life, its small scale, and its often non-commodified nature because it occurs in backyards and community gardens [10]. The exchange value of vacant land often trumps its use value for food production, limiting access as municipalities hold land for ‘higher and better use’ [11]. There is a need for higher investment in integrated city planning, effective policy portfolios, and financial mechanisms to support urban agriculture and related business enterprises. Contamination from historical land uses can require substantial investment in alternative production systems such as cap-and-fill and hydroponic systems to protect human health [12]. Urban agriculturalists may lack the technical knowledge required to grow food safely and sustainably in the city, partly due to a dearth of experimental research on best practices for urban production [4]. Urban farmers may further lack the experience and training needed to run a successful business.

Supporting urban growers and helping them to overcome these challenges is an emerging priority at the 112 land-grant universities in the United States of America (USA). These federally supported universities conduct agricultural research and provide technical training and education to growers, with a traditional focus on rural producers, either directly or through Cooperative Extension (i.e., a national educational system operated through land-grant universities in partnership with governments) [13]. It is essential to support urban agriculture through research and by creating knowledge and expertise among city residents through extension services, much like rural agricultural extension. Urban agriculture lags far behind rural agriculture in research, education, and outreach support from universities, so less is known about the needs of urban growers in the USA. Assessing the practices and needs of urban growers is an important step in the Cooperative Extension program development model [14]. Assessments of urban agriculture have often been conducted within localized areas of the USA (e.g., [15–23]) and survey questions, methods, and goals vary across assessments. At a larger scale, Census of Agriculture data on urban agriculture was analyzed [24], but many urban growers do not participate in the Census of Agriculture [23]. Two national studies surveyed and interviewed commercial urban growers [9, 25]. In a national, mixed-methods study of urban and peri-urban farmers [25], respondents reported a moderate to high need for technical assistance across a broad range of topics, from business planning to the mitigation of environmental contamination. A case study approach with 14 successful urban farmers and 150 service providers was used to understand conditions under which commercial urban farms could thrive [9]. Recommendations for actions by educators, policy makers and researchers in these two national studies focused only on commercial operations. Further study is needed at all geographic scales to incorporate the needs of nonprofits and community-focused urban farmers and gardeners and gain a more holistic view of urban agriculture.

To fill the gap in understanding the needs of all stakeholders in urban agriculture, including nonprofits and community-focused urban gardeners, and to establish a profile of such stakeholders and their organizations, we surveyed a spectrum of urban growers primarily in the northeastern USA through a collaboration among land-grant universities. Our goals were to 1) develop a profile of growers and associated organizations, 2) assess the current state of urban agriculture, and 3) determine how Cooperative Extension and research could help meet their needs.

## 2. Methods

### 2.1. Sample

Respondents were adults ages 18 and older and from the Northeast census region (which includes nine states: Connecticut, Maine, Massachusetts, New Hampshire, New Jersey, New York, Pennsylvania, Rhode Island, Vermont) in the USA, plus Delaware, Maryland, Virginia, and Washington, DC from the South census region. For simplicity, we collectively refer to the area surveyed as the Northeastern USA. We selected this geographic area because it is the most densely populated area in the USA with over 66 million people [26], it has long been recognized as a rapidly urbanizing region with interconnected population centers [27], and urban agriculture is popular within its cities. The sample consisted of 394 individuals providing valid responses to an online survey. Each question in the survey was optional, so the number of responses to a question may be lower than the total number of respondents. The sample size is indicated in the results when the number of responses is lower than the total respondents. Percentages were then calculated based on the number of valid cases, i.e., the number of respondents selecting/reporting at least one response for a question.

### 2.2. Survey instrument

The survey consisted of 47 multiple-choice and short answer questions (S1 Table), some of which were adapted from previous work [16, 25]. We refined the draft survey in two steps. In step one, we conducted cognitive interviews [28] with four urban farmers, with the goal of determining whether questions made sense in reference to the practice of urban farming and the career experience of urban farmers. Two of these interviews were conducted in-person, and two were conducted by phone or video call with farmers from Washington, DC and Baltimore, MD. The interviewer (MJ Thompson) provided participants with a copy of the survey. Participants verbally answered two practice questions to become familiar with the process and then read survey questions aloud and discussed their thought processes and answer choices. The interviewer occasionally probed for additional thoughts. We interviewed only four farmers because of the time commitment to conduct cognitive interviews and the declining return of helpful new information with each subsequent interview. We revised the survey based on feedback from cognitive interviews. In step two, we had survey development consultants with Qualtrics International (Provo, Utah, USA) review the survey, with the goal of refining questions to ensure readability and good survey practice.

We grouped questions into three main parts. The first part asked respondents for information about their urban agricultural entity such as organizational goals and challenges, number of staff, annual income and expenses, size of property used for agriculture, type of property access agreements for their site(s), products grown or produced, and their organizational role. A series of questions about aquaponic/hydroponic operations were displayed only for respondents who indicated that they engage in these operations. The second part asked respondents about their individual backgrounds and training experiences, including experience with trainings offered by Cooperative Extension, other resources used for training or information, what forms of training they found most accessible, and desired topics for future trainings. The third part asked respondents for demographic information, including age, gender, race, ethnicity, years of experience farming or growing, educational experience, and work experience in other areas besides farming.

### 2.3. Survey methods

Survey methods were reviewed and approved by University of Maryland Institutional Review Board for Human Research (IRB# 1013685–1). Qualtrics emailed individually trackable survey links to urban growers in the Washington, DC, Baltimore, and Rhode Island areas, known to us from our experiences working in these areas. Urban growers were defined as any individuals growing crops in urban areas. We, and colleagues at land-grant universities in the Northeastern USA and with other organizations, also shared an anonymous survey link through various newsletters, listservs, and social media channels. Respondents self-identified as urban or peri-urban growers, nursery workers, landscapers, government or nonprofit employees, or rural growers. To incentivize participation, the first 125 eligible respondents could elect to receive a gift card. Due to how survey invitations were distributed (email, peer-to-peer shared advertising, other postings of links to the study), it was impossible to determine a response rate.

Surveying was from 11 June through 5 September 2020. This period overlapped with the COVID-19 pandemic in the USA, resulting in a public health emergency in the geographic area included in our sample. However, responses to many questions reflect pre-pandemic information because they ask about data from 2019.

### 2.4. Data cleaning and analysis

Respondents were excluded if they self-identified as a rural grower, failed to pass a captcha (“are you human?”) type challenge, lived outside the target geographical region, or entered a string of nonsense data into responses (e.g., a random series of characters). Responses were recoded if the respondents used the “other” option to provide a written response, but their response fit one of the pre-defined responses for that question. Also, we sometimes recoded respondents’ answer about whether they engaged in hydroponics and/or aquaponics if there was a mismatch between the response to this question and their answers to the rest of the hydroponic and aquaponic questions (i.e., indicating they did not engage in one form of soilless production, but their detailed responses showed they did or vice versa). Microsoft Excel and Access (Microsoft, Washington, USA) were used to generate frequencies for the analyses.

## 3. Results

### 3.1. Respondent characteristics

A total of 394 respondents from 12 states and the District of Columbia (Table 1) completed at least some portion of the survey, with the level of missing data for individual questions increasing from 0% for the first few questions to approximately 23% for the final questions collecting race/ethnicity data. Self-reported gender for the sample was almost evenly split between male and female (Table 1). Respondent ages ranged from 18 years old to more than 75, with a plurality of respondents 35 to 44 years old and a large majority (76%) 25 to 54 (Table 1). Most respondents identified as white (Table 1) and only 10% identified as Hispanic or Latino. Overall, educational attainment was high across the sample, with 74% of respondents reporting they had completed at least four years of college or an advanced degree and all but 2% had some education beyond high school (Table 1). Respondents studied a wide range of topics in school, from Biblical studies to art to agricultural science or management, with a plurality (25%) reporting they had studied agricultural science or management. Most respondents (74%) had prior experience working in landscaping, gardening, or a plant nursery. Of the 258 respondents that reported their years of experience farming or growing (as a primary occupation), most had more than six years of experience and 38% had five or fewer years of experience (Table 1).

**Table 1:** Sociodemographic characteristics of respondents to a survey of urban growers in the Northeastern United States (n = 394).

Characteristic	n (%)*
State	
Connecticut	21 (5%)
Delaware	7 (2%)
Maine	8 (2%)
Maryland	102 (26%)
Massachusetts	49 (12%)
New Hampshire	9 (2%)
New Jersey	18 (5%)
New York	32 (8%)
Pennsylvania	36 (9%)
Rhode Island	7 (2%)
Virginia	24 (6%)
Vermont	13 (3%)
Washington, DC	62 (16%)
Multiple states	2 (1%)
Unknown	4 (1%)
Gender	
Female	152 (48%)
Male	160 (50%)
Non-binary	6 (2%)
Age	
18–25	11 (3%)
25–34	77 (24%)
35–44	108 (33%)
45–54	62 (19%)
55–64	38 (12%)
65–75	26 (8%)
75 and over	3 (1%)
Race	
American Indian/Alaska Native	9 (3%)
Asian (includes India & Middle East)	9 (3%)
Black/African American	59 (19%)
Two or more races	13 (4%)
White	213 (70%)
Highest level of education attained	
High school graduate/GED	5 (2%)
Some college	33 (10%)
Two-year college	46 (14%)
Four-year college	152 (47%)
Master’s	74 (23%)
Doctorate	14 (4%)
Years in farming as primary occupation	
Less than one year	12 (5%)
1–5 years	86 (33%)
6–10 years	93 (36%)
11–20 years	56 (22%)
More than 20 years	11 (4%)

*As a percentage of valid (non-missing) cases.

### 3.2. Organization characteristics

Respondents most frequently reported that the entity with which they were affiliated (hereafter referred to as their organization) was an urban farm, home garden, or community garden (see Table 2 for supporting data for this paragraph). Most respondents were associated with a non-profit or not-for-profit, followed by a for-profit or a hybrid organization encompassing for-profit and non-profit entities (Table 2). A crosstabulation of the most frequently reported organization types (urban farm, home garden, community garden, and school garden) with organizational tax status revealed that almost half of urban farms were for-profit. In contrast, a large majority of home and community gardens were reported to be not-for-profit.

**Table 2:** Organization characteristics at the respondent level for a survey of urban growers in the Northeastern United States. A hybrid organization encompassing for-profit and non-profit entities.

Organization type	Tax status				Total n (%)
	For-profit n (%)	Non-profit or not-for-profit n (%)	Hybrid n (%)	Other n (%)	
Urban farm	68 (49%)	43 (31%)	28 (20%)	0 (0%)	139 (35%)
Home garden	10 (11%)	58 (66%)	3 (3%)	17 (19%)	88 (22%)
Community garden	7 (8%)	66 (79%)	5 (6%)	6 (7%)	84 (21%)
School garden	2 (7%)	22 (79%)	3 (11%)	1 (4%)	28 (7%)
Landscaping and/or urban farm/garden installation service	11 (55%)	1 (5%)	8 (40%)	0 (0%)	20 (5%)
Nursery propagation	7 (58%)	3 (25%)	2 (17%)	0 (0%)	12 (3%)
Chef/restaurant garden	2 (67%)	1 (33%)	0 (0%)	0 (0%)	3 (1%)
Government agency/department	0 (0%)	2 (67%)	(0%)	1 (33%)	3 (1%)
Other:	5 (31%)	10 (63%)	1 (6%)	0 (0%)	16 (4%)
**Total**	112 (28%)	206 (52%)	50 (13%)	25 (6%)	393 (100%)

#### 3.2.1. Resource use

Agricultural production requires inputs: land, labor, water, energy, and other material resources. When asked to identify the three categories of expenses that contributed most to their organization’s annual expenses, 72% of respondents selected growing supplies (e.g., compost, fertilizer, seed) followed by labor (37%), mechanical equipment (e.g., tractors, tools) (35%), land rent or mortgage (25%), fuel and shipping (21%), land taxes (16%), marketing (e.g., farmers’ market fees, advertising) (16%), and hydroponic equipment (e.g., lights, trays) (13%).

The area farmed by organizations ranged from small to large: less than 500 square feet (19% of respondents); 500–999 square feet (18%); at least 1,000 square feet but less than 1 acre (28%); between 1 and 2 acres, 14%; between 2 and 10 acres, 15%; more than 10 acres, 5%. Most respondents (58%) reported that this area was distributed across two or more sites. Land tenure for these sites varied in terms of security and formality. When asked to indicate the types of property access agreements held for their UA sites, 12% of respondents said it was protected by a land trust, 18% had a verbal agreement, 37% had a written lease of one or more years in length with the property owner, and 38% owned the land. Most respondents (58%) rated the security of their long-term access to the property as relatively secure, selecting 4 or 5 on a five-point scale from very insecure (1) to very secure (5), with only 13% selecting a rating of 1 or 2.

Organizations relied on permanent, temporary, paid, and unpaid pools of labor. Most respondents (60%) reported their organizations employed two or more full-time staff, with 57% reporting at least two permanent, year-round staff. A total of 10 respondents (4%) reported their organizations employed 100 or more full-time staff. Volunteers, interns, and apprentices were frequently reported to be part of the organization’s labor force: of the 355 respondents reporting staffing data, 51% reported that their organization employed at least one volunteer, intern, or apprentice.

Water for production was reported to come from diverse sources, including municipal or community piped water (71% of respondents reporting at least one source), rainwater (51%), groundwater or well water (38%), or surface water (23%). Two or more sources of water were reported by 61% of respondents reporting any water source. An equally diverse array of energy sources was reported, with major sources including electricity from the power grid (66% of respondents reporting at least one source), passive solar such as a greenhouse (28%), compost as a source of heat (22%), solar photovoltaic cells (21%), gasoline (19%), solar thermal hot water (18%), and natural gas (18%). Of the 394 respondents, 78% reported using at least one energy source and 48% reported using two or more energy sources.

#### 3.2.2. Products and services

Organizations generated a wide range of products and services in 2019, with an exceptionally large majority of respondents (93%) reporting that their organizations grew food crops including vegetables (79%), herbs and spices (62%), fruit (55%), mushrooms (10%), and/or grain (10%). Cut flowers (31%) and ornamental or nursery plants (23%) were commonly grown non-food crops. A relatively small percentage of respondents reported that their organizations produced animals (11%) or honey (10%). Value-added products were produced by a small minority of organizations (12%) according to respondents. Services, including educational services (35%), landscaping/installation (20%), agritourism (19%), and catering (5%), complemented plant and animal products.

In-ground production, in beds, rows, or fields, was the most frequently reported method for producing crops (68%), followed closely by raised beds/garden boxes (64%), and containers or pots (55%). Use of greenhouses, high tunnel/hoop houses, indoor spaces, green roofs, and hydroponic, vertical, or aquaponic systems for production was less frequently reported, ranging from 28% (greenhouses) to 10% (aquaponics). In terms of certifications associated with production, 41% of respondents said their organization was certified in Good Agricultural Practices; 27% in Organic; 25% in Naturally Grown; 38% in Good Handling Practices; and 32% in Private Pesticide Application.

The distribution of organizational gross revenue (from all sources related to urban agriculture) for 2019 was strikingly bimodal, with 36% of respondents reporting less than $2,499 in net income and 21% reporting $100,000 to $999,999. Of the 295 respondents reporting revenue data, 29% reported that their organization operated at a net loss. The top three sources of income for respondents reporting any income sources were vegetable sales (52%), fruit sales (36%), grants (29%), and other agricultural products (26%). Classes, workshops, tours, demonstrations, and entertainment events were also common sources of income, reported by 25% and 16% of respondents, respectively. Of respondents, 44% reported that 100 or more individuals had participated in classes, workshops, tours, or demonstrations sponsored by their affiliated organization in 2019.

#### 3.2.3. Organizational goals and plans

Asked to choose their top three goals for their organization from a list of 14 options, respondents from for-profit and non-profit organizations most frequently selected “contribute to environmental sustainability,” “provide food for myself and/or my family,” and “increase food access and food security for my neighbors and community” (Table 3). Similarly, respondents from hybrid or other organizations also most frequently selected “contribute to environmental sustainability,” “provide food for myself and/or my family,” but then their third goal was to “build community” or “educate youth,” respectively. Revenue-related goals, including “earn a living” and “Provide supplemental income,” were not frequently selected by respondents (Table 3). Over half of respondents reported their organization had a pest management plan (59%), mission statement (58%), or food safety (53%) plan, whereas 50% or fewer reported having an emergency (38%), business (50%), marketing (46%), nutrient management (42%), conservation (38%), or farm transition or estate (28%) plan.

**Table 3:** Frequency (number and percentage) with which goals were selected as one of the top three goals for their farm, garden, or organization, by tax status, by respondents to a survey of urban growers in the Northeastern United States (n = 385).

Goals	Tax Status				Total
	For-profit	Non-profit or not-for-profit	Hybrid	Other	
Contribute to environmental sustainability	49 (45%)	91 (45%)	21 (43%)	10 (40%)	171 (44%)
Provide food for myself and/or my family	42 (39%)	82 (40%)	15 (31%)	10 (40%)	149 (39%)
Increase food access & food security for my neighbors & community	41 (38%)	67 (33%)	14 (29%)	6 (24%)	128 (33%)
Build community	30 (28%)	63 (31%)	17 (35%)	7 (28%)	117 (30%)
Increase fruit & vegetable consumption in my community	38 (35%)	43 (21%)	10 (20%)	6 (24%)	97 (25%)
Increase food & health literacy in my community	17 (16%)	49 (24%)	14 (29%)	8 (32%)	88 (23%)
Educate youth	16 (15%)	47 (23%)	9 (18%)	9 (36%)	81 (21%)
Earn a living	25 (23%)	31 (15%)	10 (20%)	6 (24%)	72 (19%)
Provide supplemental income	19 (18%)	29 (14%)	11 (22%)	2 (8%)	61 (16%)
Advocate for food justice	11 (10%)	34 (17%)	8 (16%)	5 (20%)	58 (15%)
Provide job training & business incubation for others	14 (13%)	21 (10%)	7 (14%)	3 (12%)	45 (12%)
Create safe places & reduce blight in my community	9 (8%)	20 (10%)	7 (14%)	1 (4%)	37 (10%)
Employ my neighbors & community	8 (7%)	14 (7%)	4 (8%)	0 (0%)	26 (7%)
Other	4 (4%)	19 (9%)	0 (0%)	3 (12%)	26 (7%)

*As a percentage of valid (non-missing) cases. Percentages sum to 300% because each respondent selected their top 3 goals.

#### 3.2.4. Barriers

Asked to select the top three barriers or challenges faced by their organization from a list of 13 response options, the response selected by respondents from all types of organizations was available land (all supporting data for this paragraph are in Table 4). All organizations, except for for-profits, also selected threats to long-term land access. Respondents from for-profit organizations selected two additional economic barriers: balancing a living wage for farmers with selling affordable food, and access to credit and financing. Respondents from non-profits additionally selected labor. Respondents from other organizations additionally selected regulations, zoning, and building codes. Respondents from hybrid organizations listed five barriers/challenges that tied for the most selections, including the two previously mentioned and balancing a living wage for farmers with selling affordable food, food safety compliance, and how to start a business.

**Table 4:** Frequency (number and percentage) with which respondents selected barriers/challenges as one of the top three for their organization in a survey of urban growers in the Northeastern United States, by organization tax status (n = 378).

Barrier or challenge	Tax status				Total
	For-profit	Non-profit or not-for-profit	Hybrid	Other	
Available land to start soil-based farming/gardening	37 (33%)	70 (36%)	19 (39%)	13 (54%)	139 (37%)
Threats to long-term land access	29 (26%)	65 (34%)	14 (29%)	9 (38%)	117 (31%)
Balancing a living wage for farmers with selling affordable food	41 (37%)	36 (19%)	15 (31%)	6 (25%)	98 (26%)
Labor	26 (23%)	56 (29%)	8 (16%)	4 (17%)	94 (25%)
Regulations, zoning, & building codes	21 (19%)	53 (27%)	5 (10%)	9 (38%)	88 (23%)
Water access	13 (12%)	53 (27%)	8 (16%)	6 (25%)	80 (21%)
Understanding how to navigate local bureaucracy	20 (18%)	48 (25%)	7 (14%)	4 (17%)	79 (21%)
Crop production information	23 (21%)	33 (17%)	10 (20%)	7 (29%)	73 (19%)
Access to credit & financing	30 (27%)	28 (15%)	12 (24%)	3 (13%)	73 (19%)
Food safety compliance	18 (16%)	26 (13%)	14 (29%)	0 (0%)	58 (15%)
Information on how to start a business	17 (15%)	17 (9%)	14 (29%)	6 (25%)	54 (14%)
Marketing assistance	19 (17%)	23 (12%)	8 (16%)	0 (0%)	50 (13%)
Available rooftop or building space to rent or purchase	16 (14%)	15 (8%)	6 (12%)	0 (0%)	37 (10%)
Livestock production information	10 (9%)	5 (3%)	4 (8%)	0 (0%)	19 (5%)
Legal assistance	12 (11%)	4 (2%)	1 (2%)	0 (0%)	17 (4%)
Other	4 (4%)	44 (23%)	2 (4%)	5 (21%)	55 (15%)

*As a percentage of valid (non-missing) cases. Percentages sum to 300% because each respondent selected their top 3 barriers/challenges.

#### 3.2.5. Aquaponic/hydroponic production

A total of 65 respondents indicated they used hydroponic (n = 46) and/or aquaponic (n = 38) systems in 2019 and were directed to answer supplemental questions about those systems. When asked to indicate locations of their systems, 55% reported they were in a greenhouse or high tunnel, 48% reported on a rooftop, 43% reported they were inside a building, 20% reported outdoors, and 12% reported at their home. The three most commonly grown crops were head lettuce or leafy greens (60%); peppers, tomatoes, or eggplant (40%); and herbs (38%). Vertical towers (51%) and nutrient film technique (49%) were the most commonly reported production systems used to grow these crops, followed by media beds (38%), rafts (37%), Dutch buckets (29%), and wicking beds (20%). Overall, respondents reported that they were knowledgeable about operating those systems, with most agreeing or strongly agreeing that they were knowledgeable about maintaining the pH of an aquaponic/hydroponic system (78%), diagnosing plant nutrient deficiencies (75%), and managing plant pests and diseases effectively (73%). Of those managing aquaponic systems, most agreed or strongly agreed that they were knowledgeable about diagnosing fish diseases and parasites (61%) or tracking fish growth rates (74%).

The respondents using aquaponics systems reported that they most frequently raised ornamental fish (50%) in those systems, followed by tilapia (42%), and catfish (32%), with other species reported at lower frequencies. The most commonly reported fish feeds were feed pellets (71% of respondents); aquatic plants (58%); and live feed such as insect larvae, black soldier flies, and earthworms (61%). At 68%, potassium was the nutrient supplement reported to be most frequently used in aquaponic systems, followed by iron (58%), phosphorus (53%), calcium (47%), and magnesium (21%).

### 3.3. Past experience with Cooperative Extension and future training needs

A large majority of respondents (78%) reported that they had attended Cooperative Extension training. When asked about the relevance of that training to their organization, 46% of respondents reported that all training was relevant, 42% reported that more than half was relevant, and 11% reported that less than half was relevant. When asked to rate the usefulness of eight non-Extension sources of training and information, respondents most frequently rated other farmers (49%), web resources (46%), and books (40%) as most useful.

Respondents were asked in an open-ended question to identify training topics they would like Cooperative Extension to offer urban farmers and composting (18%) and pest management (12%) were the most frequently reported training needs. Respondents were also asked to select the three topics they would most like to learn more about from a list of 22 response options and there was wide variation in responses both within and across types of organizations. Respondents from for-profits most commonly asked for training about farm finances, market opportunities, specialty crop production, and farm equipment operation, safety, and maintenance (Table 5). Respondents from non-profit and hybrid organizations most commonly asked for training on pest management for organic and certified naturally grown farms and urban soil management. Non-profits also were interested in composting, whereas seedling propagation was the third most selected topic by hybrid organizations (Table 5). Respondents from other organizations most commonly selected specialty crop production, farm/garden design, and seedling propagation (Table 5).

**Table 5:** Frequency (number and percentage) with which respondents selected additional training topics as one of their top three choices in a survey of urban growers in the Northeastern United States, by organization tax status (n = 331).

Additional training topic	Tax status				Total
	For-profit	Non-profit or not-for-profit	Hybrid	Other	
Pest management for organic & certified naturally grown farms	19 (18%)	43 (27%)	16 (33%)	2 (11%)	80 (24%)
Urban soil management (contamination, compaction, etc.)	15 (15%)	44 (27%)	10 (21%)	2 (11%)	71 (21%)
Specialty crop production (herbs, cut flowers, ethnic and cultural heritage crops, unusual fruits, etc.)	20 (19%)	28 (17%)	7 (15%)	9 (50%)	64 (19%)
Farm/garden design	13 (13%)	34 (21%)	9 (19%)	8 (44%)	64 (19%)
Seedling propagation	11 (11%)	31 (19%)	10 (21%)	6 (33%)	58 (18%)
Composting	11 (11%)	36 (22%)	6 (13%)	5 (28%)	58 (18%)
Farm finances: pricing, calculating cash flow, record-keeping, etc.	26 (25%)	19 (12%)	6 (13%)	0 (0%)	51 (15%)
Water & irrigation	16 (16%)	21 (13%)	7 (15%)	5 (28%)	49 (15%)
Fostering societal & environmental benefits through urban agriculture	13 (13%)	25 (15%)	9 (19%)	2 (11%)	49 (15%)
Sustainable production	17 (17%)	23 (14%)	8 (17%)	1 (6%)	49 (15%)
Market opportunities	21 (20%)	12 (7%)	9 (19%)	2 (11%)	44 (13%)
Farm equipment operation, safety, and maintenance	20 (19%)	14 (9%)	7 (15%)	1 (6%)	42 (13%)
GAPs or other food safety certification	11 (11%)	21 (13%)	6 (13%)	1 (6%)	39 (12%)
Food production in an unreliable climate	6 (6%)	25 (15%)	5 (10%)	2 (11%)	38 (11%)
High tunnel construction / management	12 (12%)	16 (10%)	3 (6%)	3 (17%)	34 (10%)
Data collection and analysis	15 (15%)	13 (8%)	5 (10%)	0 (0%)	33 (10%)
Harvesting techniques	10 (10%)	19 (12%)	2 (4%)	0 (0%)	31 (9%)
Biointensive production	9 (9%)	15 (9%)	4 (8%)	1 (6%)	29 (9%)
Aquaponics and/or hydroponics	8 (8%)	18 (11%)	3 (6%)	0 (0%)	29 (9%)
Vertical farming	12 (12%)	13 (8%)	2 (4%)	2 (11%)	29 (9%)
Rooftop farming	9 (9%)	11 (7%)	6 (13%)	1 (6%)	27 (8%)
Automation	15 (15%)	5 (3%)	4 (8%)	1 (6%)	25 (8%)

*As a percentage of valid (non-missing) cases. Percentages sum to 300% because each respondent selected their top three topics.

Respondents reported online training and information resources (class or workshop, article, newsletter, or video or webinar) were easier to access than in-person instruction or one-on-one consultation but also reported that they learned best from in-person classes or workshops or one-on-one consulting. Most respondents reported they had regular internet access with sufficient internet speed to watch videos (88%), but only 48% had a smartphone and 18% indicated they had slow or unreliable internet connection. Respondents most commonly reported Facebook (64%), YouTube (53%), Instagram (38%), and Twitter (34%) as the social media platforms they used for training or information about urban agriculture and business ownership.

## 4. Discussion

### 4.1. Profile of urban growers and associated organizations

Assuming participants in this survey are representative of urban growers throughout the Northeastern USA, then most are white (non-Hispanic) and younger than 45 years old. Women and men are in almost equal proportion. Urban growers are well-educated, having largely received a four-year college degree or advanced degree, but most did not receive a degree in agriculture. In lieu of a relevant college degree, most urban growers are receiving on-the-job training and have prior experience in landscaping, gardening, or plant nurseries and now identify as urban farmers/growers/producers or urban gardeners/homesteaders. However, it is possible that our methods influenced the results and the participants are not wholly representative of urban growers in the Northeastern USA. If we expect demographics of participants to reflect the demographics of the Northeastern USA, where Black people comprise 16% of the population, Native people comprise 0.1%, and Asian people comprise 3%, then the former two racial groups are overrepresented and Asian participants are underrepresented [29]. There is also a large discrepancy in educational attainment by survey participants compared to the overall population in the Northeastern USA. Only 2% of survey participants had concluded their education with a high school diploma, or had not finished high school, compared to 35% of the population in the Northeastern USA [29]. Also, 74% of survey participants had a four-year college degree or advanced degree compared to 41% of the population in the Northeastern USA [29]. The variations we see in our demographic results compared to the population of the Northeastern region could mean that certain groups, especially those with less education, were not likely to have access to the survey or complete it.

Urban agriculture in our study area was dominated by relatively small non-profit organizations operating across two or more sites often totaling only between 1,000 square feet and one acre (0.01 to 0.4 ha). Organizations were diverse in how they self-identified, with only approximately one-third identifying as an urban farm. If home and community gardens are considered similar and combined, then urban gardens would be the most common type of organization (43%). Despite the diversity of organizations represented in the survey, the most selected organizational goals largely did not differ across organizations with a different tax status. Urban agricultural organizations want to improve environmental sustainability and socio-cultural conditions through food access and security. Economic goals were not as highly prioritized.

### 4.2. Current state of urban agriculture

While a few respondents reported high gross revenue for the organizations with which they were affiliated, many reported low revenue. Diversifications of income streams—many respondents reported their organizations offered tours, classes, courses, workshops, and other activities at their site, sold products, and applied for grants—may help organizations economically and may make them more resilient. However, after factoring in growing supplies (the major expense), labor, and other expenses, most respondents reported that their organizations operated at a net loss in 2018. Whereas this is a bleak economic outlook, many organizations prioritized social and environmental goals and outcomes, which are harder to quantify and were not thoroughly captured by our survey. This economic result is also likely due to most organizations being small urban gardens that are not operated like a business.

Urban growers face diverse barriers and challenges, some unique to the urban operating environment and others found across agriculture in general, including finding access to credit and financing, labor, information on how to start a business, a balance earning a living wage for themselves and employees with selling affordable food, and information about food safety compliance. The most ubiquitous barriers and challenges reported by respondents from all types of organizations, regardless of their tax status, were related to availability of land and long-term access in urban areas, which has similarly been reported in other studies [10, 30]. Even though most respondents reported their long-term access to their current property as relatively secure, respondents from all organizations, except for-profits, still listed this as one of their top three barriers or challenges and expressed concerns about continuing access. Land availability was an even larger concern. Space is often available, even in high-density cities [31], but its use for agriculture may be limited due to regulations, zoning, building codes, or costs.

The types of spaces available may dictate the water and energy sources used for urban agriculture. Urban agriculture may rely on potable municipal or community water [32] or increase use of energy within cities [33], which has economic, socio-cultural, and environmental costs and may reduce the sustainability and resilience of urban gardens and farms. Urban production systems are largely soil-based systems, which have lower start-up costs than controlled environment systems. Soil-based systems may be more environmentally friendly than indoor soilless systems, particularly if the indoor systems increase grey infrastructure and have high energy demands. However, turnover of crops is typically higher in indoor soilless systems, which often allow for year-round production. Additionally, indoor systems have fewer pest and disease problems, and may have lower demands for labor, water, and nutrients per unit area of growing space since they can be automated and recycle water and nutrients.

Our results suggest that there are relatively few urban aquaponic systems in the Northeastern USA compared to other common urban production systems. The current urban aquaponic producers can do more to raise revenue, be more environmentally sustainable, and benefit urban consumers, but there can be a trade-off between economic benefits and environmental and social benefits. Currently, aquaponic (and hydroponic) systems occupy diverse urban spaces, particularly greenhouse/high tunnels, rooftops, and building interiors. These systems largely produce leafy greens, which generate lower revenue per ounce than other possible crops but have high turnover yielding high returns on an annual basis and are in demand by the local community. Ornamental fish are the most common in our study area. They are common in aquaponics, although in most cases they are second to tilapia in their use [34, 35]. Urban growers may raise ornamental such as koi because they generate greater revenue than edible fish such as tilapia. Producing tilapia could benefit consumers in urban neighborhoods with limited access to food and protein sources, but aquaponic growers struggle to sell tilapia at a price that covers their costs of production [36]. Also, ensuring that energy is generated from renewable sources, using duckweed or insect larvae as fish food to replace or supplement purchased dry pellets [37], and maximizing the use of fish waste for crop production [38] can help increase environmental benefits.

### 4.3. The role of Cooperative Extension and research

Although most respondents had attended training offered through Cooperative Extension, they identified a wide range of additional training needs, and the training needs differed depending on the tax status of the respondent’s organization. Some training needs are relatively standard and met by current Cooperative Extension trainings, such as soil management, pest management, composting, seedling propagation, and specialty crop production, which may at most need to be adapted to meet urban growers’ specific needs. Perhaps the most effective first step in outreach to urban growers by Cooperative Extension would be to improve the marketing of existing training opportunities, offering information at times and through methods most convenient and effective for urban growers. We additionally recommend a series of financial management and business trainings targeted specifically to the needs of urban growers, especially those at for-profit organizations, to help them navigate market opportunities and farm finances. Some other primary opportunities for Cooperative Extension are to help urban growers understand and draft a mission statement and business, operational, and emergency plans and obtain certifications. Cooperative Extension targeting rural farmers already provides training related to marketing and business management, drafting mission statements and plans, and opportunities to obtain certificates. The adaptation of these resources, or creation of a collaborative educational community, could support the diverse educational needs of for-profit urban farmers. Long term connection among small scale rural farmers and urban farmers would offer new networks that support longer-term growth and collaborations that serve both. Whereas respondents noted that online training was easier to access, in-person training was better for learning, so in-person training should especially be the emphasis for urban growers that come from an academic background not related to agriculture. Several urban growers had experience in journalism, media, the arts, television, and internet, so these individuals might be extremely helpful in identifying ways that universities can better connect with urban growers and for sharing their personal experience in urban farming with others through a train-the-trainer program.

Soilless production is surprisingly limited in urban spaces, according to the study results, so training in hydroponic and aquaponic systems and their benefits is an opportunity for increased outreach. Whereas aquaponics can require more up-front costs [39] and technical proficiency, there are noted benefits for urban environments and communities, including more efficient water use and providing a lean source of fish protein to urban residents. Urban growers using aquaponic production systems self-reported their skills as being relatively high, but less than half had skill diagnosing fish diseases or parasites and only 58% had skill tracking fish growth rates. These skills are relatively important for maintaining aquaponics systems and maximizing profit from selling fish, indicating a need for investment in extension education and training in these aspects.

In addition to educational opportunities, the field of urban agriculture may benefit from research, especially pertaining to 1) pest management; 2) why people are attracted to urban agriculture, especially when their academic major was not related to agriculture, and how to attract more people, especially from minoritized communities; 3) methods for economic, social, and environmental sustainability of urban production systems; 4) policies and regulations that promote and support urban agriculture, with an emphasis on increasing access and equity for underrepresented groups; 5) using recycled water for irrigation, and alternative and environmentally friendly sources of energy; 6) identifying space available for urban agriculture (as in [31]) and threats to long-term land access; and 7) market opportunities. Whereas this research is already being implemented in some cities, results are usually dependent on local social-ecological conditions, so individual cities must have their own robust research programs [40–42]. Urban residents, especially from marginalized communities, can also have a well-founded hesitation to engage with universities and government agencies conducting research. In our experience the best way to build trust is to engage leading community members and organizations and to engage in participatory research, in which urban growers are directly involved in what, where, and how research is conducted. University researchers should also expect to provide adequate funding and labor for all research projects in urban spaces and should avoid any additional burden to urban growers.

## 5. Conclusion

Urban agriculture is becoming increasingly popular and more supported by local communities and through funding at the local, state, and federal level because of its economic, social, and environmental benefits. Universities can play a key role in promoting urban agriculture by offering training and research and our survey results help identify the current profile of urban growers and their organizations to better support them. Our survey research was conducted online in one region of the USA. Our results may not be wholly generalizable to the Northeastern USA due to fewer people with less education responding than expected. Whereas the Northeastern USA is a densely populated area of the country and urban agriculture is relatively common, the results also may not be generalizable to other regions where economic, social, and environmental conditions differ. Expanding the survey to other geographic regions may help identify broad needs and opportunities within urban agriculture, although potentially with some loss of information at the more local scale. Expanding the survey and identifying additional delivery methods, such as capturing qualitative feedback from in-person surveys, would also capture more responses from for-profit organizations and people missed by our online survey. Our results are largely indicative of the current state of urban agriculture being dominated by non-profit organizations, but the lower sample size of for-profit organizations implies that our results are less representative of these organizations.

Urban agriculture is rapidly changing and expanding due to market factors, community demand, and other external forces such as supply chain issues from political unrest, climate change, and pandemics. Workforce development is a large priority among institutions of higher education, so urban growers should regularly be consulted, and the results shared with career and workforce development professionals and researchers in urban areas to identify training and research that meets the needs of stakeholders.

## Supporting information

S1 Table Survey administered to 406 urban growers in the USA. (DOCX)

S2 Table All data underlying the findings. (XLSX)
