# Supplementary material for: A profile of urban agricultural growers, organizations, their needs, and challenges in the Northeastern United States
Source: PLoS One. 2024 Apr 10;19(4):e0298831. doi: 10.1371/journal.pone.0298831 (PMC11006126; doi:10.1371/journal.pone.0298831)
Supplement: S1 Table — (DOCX) [file pone.0298831.s001.docx]

S1 Table. Survey administered to 406 urban growers in the USA

**Part 1: Help us paint a picture of urban agriculture: Questions about your farm, garden, organization, or business.**
 Urban agriculture includes a diverse network of activities and entities: farms, gardens, landscapers, nurseries, non-profits, for-profits, schools, composters, makers of food products, consultants, governmental agencies, and more. We understand that you may participate in urban agriculture in multiple ways, for example as a community gardener and as a teacher with a school garden. But when you answer the questions in Part 1, please think of one urban agricultural entity you play the most significant role in and answer the questions with that organization in mind. This will help us understand your answers.

**Note:** For the purposes of this survey, an urban agricultural entity with multiple locations counts as one entity; for example, an urban farm that grows on four different lots around a city, or a landscaper who serves multiple clients.

Q1 In what kind of urban agricultural entity do you play the most significant role (as measured by time invested, position within the entity, or highest percentage of your personal income)?

- Urban farm
- Community garden
- Home garden
- School garden
- Chef/restaurant garden
- Nursery propagation
- Landscaping and/or urban farm/garden installation service
- Government agency/department
- Other: ________________________________________________

Q2 If your farm, garden, or organization has a name, please list the full name here. Write N/A if there is no name.

We will not keep this information; it will be converted into a numeric code to keep your answers confidential._________________________________________________________

Q3 Which one of the following statements best describes your role with your farm, garden, or organization?

- I am an urban farmer / grower / producer
- I am an urban gardener or homesteader
- I am a nursery worker / manager
- I am a peri-urban farmer / grower / producer
- I am a landscaper / urban farm/garden installer
- I am a government or non-profit employee (not primarily a grower)
- I am a rural farmer or gardener
- Other: ________________________________________________

Q4 Is your farm, garden, or organization organized primarily as a for-profit or non-profit?

- For-profit
- Non-profit or not-for-profit
- Hybrid - have both for-profit and non-profit entities as part of our organization
- Other: ________________________________________________

Q5 What are your goals for your farm, garden, or organization?
Please rank your TOP THREE by putting the number 1, 2, or 3 next to your top choices.

______ Earn a living

______ Provide supplemental income

______ Increase food access and food security for my neighbors and community

______ Provide food for myself and/or my family

______ Employ my neighbors and community

______ Provide job training and business incubation for others

______ Increase food and health literacy in my community

______ Increase fruit and vegetable consumption in my community

______ Build community

______ Create safe places and reduce blight in my community

______ Advocate for food justice

______ Educate youth

______ Contribute to environmental sustainability

______ Other:

Q6 Which of the following do you see as the biggest barriers or challenges to your farm, garden, or organization?
Please rank your TOP THREE by putting the number 1, 2, or 3 next to your top choices.

______ Available land to start soil-based farming/gardening

______ Threats to long-term land access

______ Crop production information

______ Livestock production information

______ Marketing assistance

______ Access to credit and financing

______ Legal assistance

______ Labor

______ Information on how to start a business

______ Food safety compliance

______ Available rooftop or building space to rent or purchase

______ Regulations, zoning, and building codes

______ Balancing a living wage for farmers with selling affordable food

______ Water access

______ Understanding how to navigate local bureaucracy

______ Other:

Q7 How secure or insecure do you feel about your long-term access to the property on which you farm/garden/grow?

|  | 1 | 2 | 3 | 4 | 5 |  |
| --- | --- | --- | --- | --- | --- | --- |
|  |  |  |  |  |  |  |
| Very insecure |  |  |  |  |  | Very secure |

Q8 What kind of property access agreement do you have? If you have multiple locations, please check all that apply.

- Verbal agreement with property owner
- Written use permit with property owner
- Written one year lease
- Written two to five year lease
- Written lease for more than five years
- Ownership of property
- Land protected by land trust or land bank
- Agreement with client to provide service on their property (landscaping, green roof maintenance, etc.)
- Other: ________________________________________________

Q9 What did you produce in 2019? Please check all that apply.

- Fruit (including berries, melons, and tree fruits)
- Vegetables
- Herbs and spices
- Animals
- Honey
- Cut flowers
- Mushrooms
- Ornamentals and/or nursery plants
- Grain crops
- Value-added products (jams, preserves, soaps, etc)
- Educational services
- Agritourism services
- Landscaping/installation services
- Catering services
- Other (please specify):_________________________________

Q10 Thinking of what you produced in 2019, roughly what percentage did you sell, donate, or use internally (used yourself or within your entity)?

|  | Percent |
| --- | --- |
| Sold |  |
| Donated / given away |  |
| Used internally |  |

Q11 What production systems did you use in 2019? Please check all that apply.

- Containers or pots
- Raised beds / garden boxes
- Growing in-ground in beds, rows, or fields
- Hydroponic
- Aquaponic
- Green roof
- Greenhouse (permanent rigid walls)
- High tunnel / hoop house (removable flexible plastic walls)
- Indoor (a building other than greenhouses, high tunnels, and hoop houses)
- Vertical
- Other (please specify): ______________________________________

Q12 How much space did you farm or garden in 2019?

*For reference, 500 square is about the size of 3 parking spaces. One acre is 43,560 square feet, or about the size of a football field not including the end zones.*

- Less than 500 square feet
- Between 500 and 1,000 square feet
- Between 1,000 square feet and 1 acre
- Between 1 and 2 acres
- Between 2 and 10 acres
- More than 10 acres
- I don't know

Q13 How many different growing/production sites does your farm, garden, or organization manage?

- 1
- 2
- 3
- 4
- 5
- More than 5

Q14 How many people (including yourself) are employed or volunteer on your farm, garden, or organization each year?

- Full-time employees (paid for more than 130 hours/month) ____________
- *Of these full-time employees, how many do you employ year-round?* ____________
- Part-time employees (paid for fewer than 130 hours/month) __________
- *Of these part-time employees, how many do you employ year-round?* ____________
- Volunteers _________
- Interns and apprentices _________

Q15 Please indicate the top three **sources of income** for your farm, garden, or organization in 2019.

- Sales of vegetables
- Sales of fruits (melons, berries, tree fruits)
- Sales of fish
- Sales of poultry or eggs
- Sales of other agricultural products (e.g., seedlings, compost, fertilizer, value-added products, etc.)
- Classes / workshops / tours / demonstrations
- Entertainment events (e.g., catered meals, weddings, comedy shows, movies, festivals, etc.)
- Grants
- Farm or gardening work for others
- Other (Please specify): _____________________

Q16 Please indicate the three categories that contribute the most to the **annual expenses** of your farm, garden, or organization.

- Labor
- Land rent or mortgage
- Land taxes
- Marketing (farmers' market fees, advertising, etc.)
- Fuel and shipping
- Growing supplies (compost, fertilizer, seed, etc.)
- Mechanical equipment (tractors, tools, etc.)
- Hydroponic equipment (lights, trays, etc.)
- Other: ________________________________________________

Q17 In 2019, what was your farm's or organization's **gross** income from all sources related to urban agriculture (**before** expenses)?

- Less than $2,499
- $2,500 - $9,999
- $10,000 - $24.999
- $25,000 - $49,999
- $50,000 - $99,999
- $100,000 - $999,9999
- $1 million or more
- I don't know

Q18 In 2019, what was your farm's or organization's **net** income from all sources related to urban agriculture (**after** expenses, which includes all salaries)?

- Negative net income
- $0 - $2,499
- $2,500 - $9,999
- $10,000 - $24.999
- $25,000 - $49,999
- $50,000 - $99,999
- $100,000 - $999,9999
- $1 million or more
- I don't know

Q19 In 2019, approximately how many individuals have taken part in tours, classes, courses, or workshops at your farm, garden, business, or classroom?

- None
- 1 to 24
- 25 to 49
- 50 to 99
- 100 to 499
- 500 to 999
- 1,000 to 4,999
- 5,000 or greater
- I don't know

Q20 If providing educational, therapeutic, or workforce development services is among the primary missions of your farm, garden, or organization, which groups do you primarily serve? Please check all that apply.

- Not applicable
- Pre-K youth
- K-12 youth
- Students in post-high-school programs (college, community college, etc.)
- Working-age adults
- Senior adults
- All ages
- Veterans
- Returning citizens (people who have been incarcerated)
- "Opportunity youth” (ages 16-24, not in school or employed)
- People in recovery from addiction
- People experiencing homelessness
- Job seekers in general
- Other: ________________________________________________

Q21 Please indicate whether your farm, garden, or organization has the following plans for at least one of its locations.

|  | Has | Does not have | Not relevant | I'm not sure |
| --- | --- | --- | --- | --- |
| Emergency Plan |  |  |  |  |
| Mission Statement |  |  |  |  |
| Business Plan |  |  |  |  |
| Marketing Plan |  |  |  |  |
| Nutrient Management Plan |  |  |  |  |
| Conservation Plan |  |  |  |  |
| Farm Transition or Estate Plan |  |  |  |  |
| Pest management plan |  |  |  |  |
| Food safety plan |  |  |  |  |
| Other (please specify): |  |  |  |  |

Q22 Please indicate whether your farm, garden, organization, or an employee of the farm/garden/organization, has the following certifications.

|  | Has | Does not have | Not relevant | I'm not sure |
| --- | --- | --- | --- | --- |
| Good Agricultural Practices (GAPs) |  |  |  |  |
| Good Handling Practices (GHPs) Certification |  |  |  |  |
| Private Pesticide Applicator Certification |  |  |  |  |
| Certified Organic |  |  |  |  |
| Certified Naturally Grown |  |  |  |  |
| Other (please specify): |  |  |  |  |

Q23 Which water sources did you use for urban agriculture in 2019? CHECK ALL THAT APPLY.

- Municipal or community piped water
- Groundwater or well water
- Surface water (from streams, lakes, springs, or reservoirs)
- Rainwater
- Other ________________________________________________

Q24 Which energy sources did you use for urban agriculture in 2019? CHECK ALL THAT APPLY.

- Electricity from the power grid
- Natural gas
- Propane
- Compost (as a source of heat)
- Geothermal
- Passive solar (such as a greenhouse)
- Solar thermal hot water
- Solar photovoltaic cells
- Wind energy
- Wood-burning / pellet stove or boiler
- Gasoline
- Other: ________________________________________________

Display This Question if Q11 = Hydroponic or Aquaponic

Q25 Where is your aquaponics or hydroponics system located? CHECK ALL THAT APPLY.

- Inside a building
- Inside a greenhouse or high tunnel
- On a rooftop
- Outdoors
- At your home

Display This Question if Q11 = Hydroponic or Aquaponic

Q26 What types of FISH were raised at your aquaponics facility in 2019? CHECK ALL THAT APPLY.

- Not applicable, I use hydroponics but not aquaponics
- Tilapia
- Catfish
- Bluegill
- Yellow perch
- Ornamental fish (Koi, goldfish, tropical fish)
- Bass
- Trout
- Atlantic salmon
- Other: ________________________________________________

Display This Question if Q11 = Hydroponic or Aquaponic

Q27 What types of FISH FEED did you provide in 2019? CHECK ALL THAT APPLY.

- Not applicable, I use hydroponics but not aquaponics
- Feed pellets (dry fish feed)
- Aquatic plants (duckweed, azolla, etc.)
- Live feed (insect larvae, black soldier flies, earthworms, etc.)
- Food scraps
- Other: ________________________________________________

Display This Question if Q11 = Hydroponic or Aquaponic

Q28 What types of CROPS were raised at your aquaponics or hydroponics facility in 2019? CHECK ALL THAT APPLY.

- Herbs
- Head lettuce or leafy greens (e.g. collard greens, kale, bok choi, chard, etc.)
- Peppers, tomatoes, or eggplant
- Beans or peas
- Cucumbers, melons, or squash
- Berries
- Watercress or duckweed
- Root crops
- Celery, broccoli, cabbage, or cauliflower
- Other: ________________________________________________

Display This Question if Q11 = Hydroponic or Aquaponic

Q29 Which of the following CROP PRODUCTION METHODS were used in your aquaponics or hydroponics facility in 2019? CHECK ALL THAT APPLY.

- Rafts (planting in floating trays)
- Media beds (gravel or other media filled boxes)
- Nutrient film technique (NFT, horizontal irrigated trays or gutter planters)
- Vertical towers (vertical irrigated pipe planters)
- Wicking beds
- Dutch buckets
- Other ________________________________________________

Display This Question if Q11 = Hydroponic or Aquaponic

Q30 Please indicate whether you agree or disagree with the following statements:

|  | Strongly disagree | Disagree | Agree | Strongly agree |
| --- | --- | --- | --- | --- |
| I know how to maintain the pH of an aquaponic/hydroponic system |  |  |  |  |
| I think alkalinity is an important parameter to be monitored and controlled in aquaponics /hydroponics |  |  |  |  |
| I know how to diagnose FISH diseases and parasites |  |  |  |  |
| I know how to track FISH growth rates |  |  |  |  |
| I know how to diagnose PLANT nutrient deficiencies |  |  |  |  |
| I know how to manage PLANT pests and diseases effectively |  |  |  |  |

Display This Question if Q11 = Hydroponic or Aquaponic

Q31 What NUTRIENT SUPPLEMENTS have you added to your aquaponic system in 2019? CHECK ALL THAT APPLY.

- Not applicable, I use hydroponics but not aquaponics
- Iron
- Potassium
- Phosphorus
- Calcium
- Magnesium
- Other ________________________________________________

**Part 2: Your training and experiences**For the rest of the survey, please answer the questions thinking of yourself and your own experiences, rather than anyone else at your farm/garden/organization.

Q32 To your knowledge, have you ever attended an Extension training?
*(Includes brief workshops, longer courses, and webinars taught by Extension Educators or Agents)*

- Yes
- No
- I do not know

Display This Question if Q32 = Yes

Q33 Thinking of the Extension trainings you have attended, how relevant was the training to your urban farm?

- All of it was relevant
- More than half was relevant
- Less than half was relevant
- None of it was relevant

Q34 Other than Extension, what training and information resources do you find most useful?

|  | Least useful | . | . | . | Most useful | N/A |
| --- | --- | --- | --- | --- | --- | --- |
| Private consultant |  |  |  |  |  |  |
| Distributor / aggregator / food hub |  |  |  |  |  |  |
| Veterinarians |  |  |  |  |  |  |
| Farm Bureau |  |  |  |  |  |  |
| Books |  |  |  |  |  |  |
| Web resources |  |  |  |  |  |  |
| Other farmers |  |  |  |  |  |  |
| Chefs |  |  |  |  |  |  |
| Other (please specify): |  |  |  |  |  |  |

Q35 What training topics would you want Extension to offer urban farmers?

________________________________________________________________

________________________________________________________________

Q36 Based on informal conversations with urban farmers over the past year, we are considering offering more training and educational articles on the following topics. 
Please click the box next to the THREE topics you would MOST like to learn more about.

- Pest management for organic and certified naturally grown farms
- Water and irrigation
- GAPs or other food safety certification
- Specialty crop production (herbs, cut flowers, ethnic and cultural heritage crops, unusual fruits, etc.)
- High tunnel construction / management
- Farm finances: pricing, calculating cash flow, record-keeping, etc.
- Farm equipment operation, safety, and maintenance
- Urban soil management (contamination, compaction, etc.)
- Seedling propagation
- Harvesting techniques
- Composting
- Biointensive production
- Food production in an unreliable climate
- Aquaponics and/or hydroponics
- Rooftop farming
- Vertical farming
- Market opportunities
- Data collection and analysis
- Fostering societal and environmental benefits through urban agriculture
- Automation
- Sustainable production
- Farm/garden design

Q37 What type of training is **easy for you to access**? What type of training do you think **best helps you learn**?

|  | Easy for me to access | Helps me to learn |
| --- | --- | --- |
| In-person class or workshop |  |  |
| Online class or workshop |  |  |
| Online article |  |  |
| Online newsletter |  |  |
| Online videos or webinar |  |  |
| One-on-one consulting |  |  |

Q38 Which types of Internet connections do you consistently have access to? Check as many as apply.

- Sufficient internet speed to watch videos
- Slow or unreliable internet connection
- Smartphone
- I don’t have regular internet access

Q39 Which types of social media do you use for training or information about urban agriculture and business ownership? Check as many as apply.

- Facebook
- Twitter
- Instagram
- YouTube
- Pinterest
- LinkedIn
- I do not use social media
- Other (please specify): _____________________________________

**Part 3: General information about you and your farm**
This information is used only to help us analyze the data. We do not share your individual data with any organization or person.

Q40 In what city and state do you farm, garden, or engage in urban agriculture?  ____________

Q41 Please indicate the number of years you have been farming or growing (as your primary occupation).

- Less than one year
- 1-5 years
- 6-10 years
- 11-20 years
- More than 20 years
- Not applicable - I have not had farming or growing as my primary occupation

Q42 Please indicate your gender.

- Male
- Female
- Non-binary
- Prefer not to say

Q43 Please indicate your ethnicity.

- Hispanic/Latino
- Not Hispanic/Latino
- Prefer not to say

Q44 Please indicate your race.

- American Indian/Alaska Native
- Asian (includes India & Middle East)
- Black/African-American
- Native Hawaiian/Other Pacific Islander
- White
- Two or more races
- Prefer not to say

Q45 Please indicate your age range.

- 18-25
- 25-34
- 35-44
- 45-54
- 55-64
- 65-75
- 75 and Over
- Prefer not to say

Q46 Please indicate the highest level of formal education that you have completed.

- Less than high school
- High school graduate/GED
- Some college
- Two-year college
- Four-year college
- Master’s
- Doctorate
- Prefer not to say

Q47 What was your main topic of study (or major) in school?

- Business / Management
- Education
- Engineering / Computer Science / Mathematics
- Health
- Humanities
- Law
- Life / Physical Sciences: Not including agriculture
- Agricultural science / management
- Social / behavioral sciences
- Vocational / technical
- Other: ________________________________________________

Q48 Other than farming, what type of work experience do you have? Please check all that apply.

- Landscaping, garden center, or plant nursery
- Business management or accounting
- Administrative or clerical
- Military service
- Sales or retail
- Food service
- Teaching and education
- Health care
- Construction, plumbing, or electrical
- Mechanic
- Government / policy
- Other ________________________________________________

Q49 Please share anything else with us about your urban agriculture experiences that you want us to know.

________________________________________________________________

________________________________________________________________
